# Evidence of Recombination Suppression Blocks on the Y Chromosome of Date Palm (*Phoenix dactylifera*)

**DOI:** 10.3389/fpls.2021.634901

**Published:** 2021-04-20

**Authors:** Maria F. Torres, Yasmin A. Mohamoud, Shameem Younuskunju, Karsten Suhre, Joel A. Malek

**Affiliations:** ^1^Department of Biological Sciences, University of Cincinnati, Cincinnati, OH, United States; ^2^Genomics Laboratory, Weill Cornell Medicine in Qatar, Doha, Qatar; ^3^Department of Physiology, Weill Cornell Medicine in Qatar, Doha, Qatar; ^4^Department of Genetic Medicine, Weill Cornell Medicine in Qatar, Doha, Qatar

**Keywords:** date palm, sex determination, recombination suppression, Y chromosome strata, phased sequencing

## Abstract

The genus *Phoenix* includes the fruit producing date palm tree among 14 species that are all dioecious. Females produce the fruit that are high in sugar content and used in multiple countries ranging from North Africa to South Asia, especially from the *Phoenix dactylifera*, *Phoenix sylvestris*, and *Phoenix canariensis* species. While females produce the fruit, understanding of the genetic basis of sex control only began recently. Through genus-wide sequencing of males and females we recently identified three genes that are conserved in all males and absent in all females of the genus and confirmed an XY sex chromosome system. While our previous study focused on conservation of male-specific sequences at the genus-level, it would be of interest to better understand the spread of male-specific sequences away from the core conserved male genes on the Y chromosome during speciation. To this end, we enumerated male-specific 16 bp sequences using three male/female pairs from the western subpopulation of date palm and documented the density of these sequences in contigs of a phased date palm genome assembly. Here we show that male specific sequences in the date palm Y chromosome have likely spread in defined events that appear as blocks of varying density with significant changes in density between them. Collinearity of genes in these blocks with oil palm shows high synteny with chromosome 10 between megabase 15 and 23 and reveals that large sections of the date palm Y chromosome have maintained the ancestral structure even as recombination has stopped between X and Y.

## Introduction

Date palm (*Phoenix dactylifera*) is one of the most ancient cultivated trees that have adapted to withstand extreme environmental conditions prevalent in arid and semi-arid regions. Evidence of date palm cultivation and consumption dates back to ancient Mesopotamian and Egyptian monuments which contain drawings of palms and to this day maintains a significant religious and cultural value in the Middle East and North Africa (Popenoe, 1924).

All members of the genus Phoenix including the date palm are dioecious and hybridization between different species in the genus is common [reviewed in Gros-Balthazard (2013)]. Early biotechnological interest in the sex determination system of date palm arose because males do not produce fruit and only a single male tree is needed to pollinate multiple female trees. The observation that seed propagated date palm produce equal numbers of male and female progeny suggested that sex determination was genetic and not environmental.

The first description of date palm chromosomes involved observations of root tip cells from multiple male individuals, but no particular differences in length between chromosome pairs was detected (Beal, 1937). Almost 60 years later, Siljak-Yakovlev and collaborators used a probe to identify heterochromatin in chromosome spreads and determined that females had a pair of similar heterochromatin spots while the analogous pair in males was heteromorphic, suggesting the presence of an XY sex-determination system in date palm (Siljak-Yakovlev et al., 1996).

Early insights into the anatomical features of date palm flowers suggested few morphological differences between male and female flowers during early stages of development (De Mason et al., 1982). More recent cytological studies revealed that date palm flowers are initially bisexual as indicated by the presence of carpel and stamen primordia in both male and female flowers, but during later stages of development, staminode and pistillode cells stop dividing and remain undifferentiated in female and male flowers respectively, leading to fully developed unisexual flowers (Daher et al., 2010). Exogenous application of synthetic plant hormones during these initial stages of male and female flower development lead to the production of flowers morphologically classified as bisexual, suggesting that both male and female individuals carry most of the genetic controls for male and female function (Torres and Tisserat, 1980; Masmoudi-Allouche et al., 2009). Attempts to characterize these gender determinants involved the use of isozymes and other molecular markers including RAPDs and microsatellites (Torres and Tisserat, 1980; Younis et al., 2008; Cherif et al., 2013).

In 2011, our group sequenced and released the first draft of the date palm genome (Al-Dous et al., 2011). Tests for various genetic models of sex determination allowed the identification of heterozygous single nucleotide polymorphisms (SNPs) shared by all males and absent in all females tested (Al-Mahmoud et al., 2012). This gender-linked region was later mapped to the lower arm of linkage group 12 and is estimated to span between 6 and 13 Mb (Mathew L. S. et al., 2014; Hazzouri et al., 2019). Despite the work of multiple groups, identification of sex determining genes in date palm remained elusive given the presence of a significant number of predicted gene candidates in the gender segregating regions. Phylogenetic analysis of a gender-linked locus revealed that it segregated with sex in multiple *Phoenix* species, suggesting that recombination arrest of the gender determination region, and most likely dioecy, evolved before speciation (Cherif et al., 2016). We utilized this information and a modified approach used to identify Y-linked male determination genes in persimmon and kiwifruit (Akagi et al., 2014, 2019) to identify genus-wide conserved male sequences. We searched for short sequences (kmers) present in all males but absent in all females of the genus *Phoenix* and identified three genes absent from all previously sequenced female Phoenix individuals (Torres et al., 2018).

**FIGURE 1:**
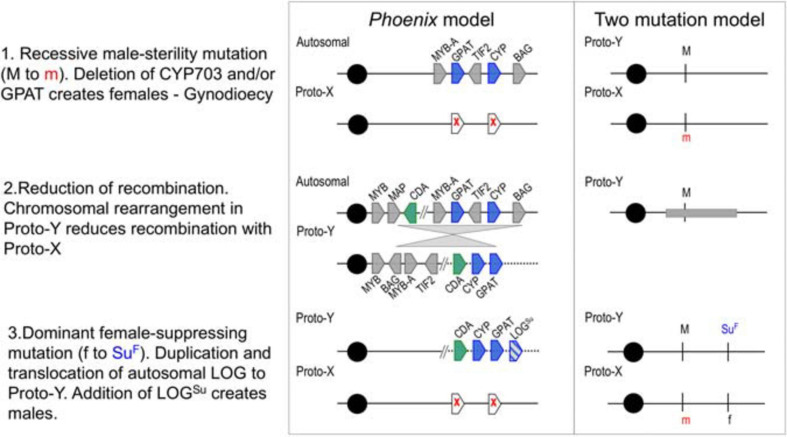
A proposed step-wise model of evolution in the *Phoenix* (including Date Palm) sex-determination region compared with the two-gene model for sex determination (adapted from Charlesworth et al., 2005). Two genes are deleted from Proto-X (white pentagons with red x), a later rearrangement (gray triangles) relocates multiple genes and a final duplication and translocation of a gene (pentagon with blue lines) with potential female-suppressing characteristics (LOG^*Su*^) creates males and females. Gene abbreviations and gene IDs MYB: LOC105059740, MAP: LOC105059742, CDA: LOC105059743, MYB-A: LOC105059783, GPAT (GPAT3): LOC105059961, TIF2: LOC105059784, CYP (CYP703): LOC105059962, BAG: LOC105059785. Cytidine deaminase (CDA) was the only gene with male-conserved kmers present in both male and female individuals.

Analysis of the evolutionary history and divergence rates of these genes, suggested that two male-sterility mutations (CYP703 and GPAT3) and an inversion event suppressed recombination in the region, leading to gynodioecy and a translocation of a female-suppressing mutation (LOG) probably established males (Figure 1). These findings support the two-mutation model for the origin of sex chromosomes (Westergaard, 1958; Charlesworth and Charlesworth, 1978). While this approach allowed the identification of the events that likely initiated the differentiation between X and Y chromosomes in *Phoenix*, it left out the gender-linked region previously identified in the date palm (Mathew S. et al., 2014; Hazzouri et al., 2019). Based on the observation of translocations and inversions in the highly conserved sex determination region, we hypothesized that the evolution of the Y chromosome in date palm may have involved multiple events even after speciation, leading to different levels of divergence across the sex chromosomes, as described in two plant species (Bergero et al., 2007; Wang et al., 2012). While our goal in the previous study was to identify conservation of sex determination genes across the genus, this study aims to identify the broadest regions of divergence between the sex chromosomes in the date palm species alone. Our previous study took advantage of male/female genome comparisons across the genus, while this study utilized the unique resource of male/female genome comparisons within a single (Western) date palm subpopulation (Mathew et al., 2015; Mohamoud et al., 2019). In so doing, we hope to provide a resource to begin understanding the process and breadth of divergence between X and Y chromosomes of date palm.

## Materials and Methods

### Sample Collection

Samples used in this manuscript have been described previously. Briefly, date palm and Phoenix male/female accessions were obtained from the USDA collection at Riverside, CA. Deglet Noor, Zaghloul and Hayani male and female sequence data was collected from the NCBI SRA trace archive using accessions: SRR6439416, SRR6439410, SRR6716026, SRR6716027, SRR6716028, and SRR6716029.

### Genome Sequencing

The 10X phased genome data was utilized from our previous study. Briefly, a Deglet Noor male that was backcrossed for five generations (DNBC5) at the USDA collection in California was used. DNA was extracted and sequenced according to the 10X Genomics recommended protocol for sequencing genomes with 1 ng of input DNA. Phasing of the two alleles was conducted using SUPERNOVA v1.1 with standard settings (10X Genomics, CA, United States).

### Data Analysis

**FIGURE 2:**
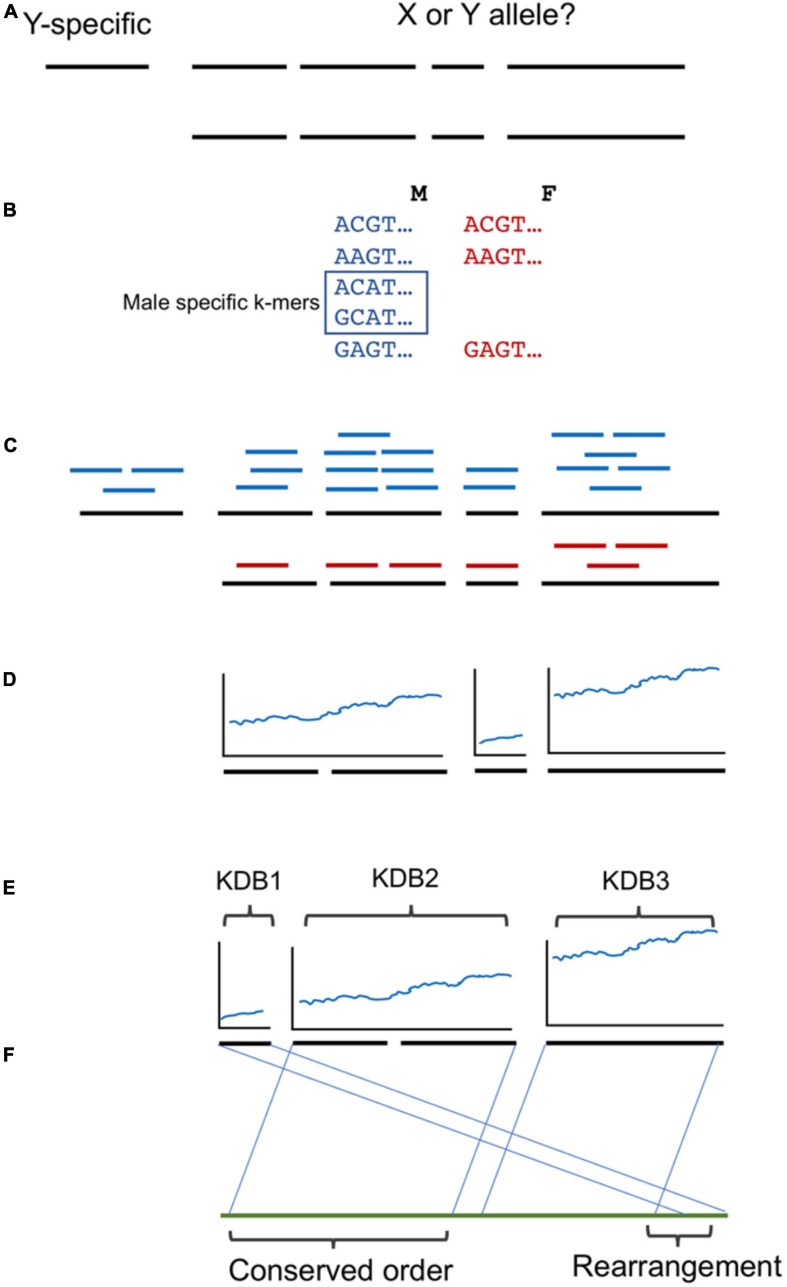
Workflow used in this study. **(A)** A phased male date palm genome was used as reference. While Y specific contigs are clear by the absence of a second allele, which alleles are X or Y linked for the remainder is unclear. **(B)** Three male/female pairs of the western date palm subpopulation were used to identify male-specific 16 bp k-mers. **(C)** Contigs with one allele showing higher density of male-specific k-mers were selected as Y-linked. **(D)** The density of male specific k-mers was noted and **(E)** groups of contigs ordered by blocks of shared k-mer density (KDB). **(F)** The ordering was compared to oil palm and conserved ordering or rearrangements noted.

A brief overview of the workflow and analysis is provided in Figure 2. We utilized a similar kmer approach as described previously (Torres et al., 2018). Briefly, kmers of 16 base pairs in length were collected from the FASTQ read files of each genome using JELLYFISH v1.1.11 (Marçais and Kingsford, 2011). A histogram of the counts of the 16 bp kmers was plotted to determine the average coverage for each genome and the trough of kmers containing sequencing errors. All kmers present in the Deglet Noor male more than 24 times were included as possible male or female kmers, while kmers present in the Deglet Noor female greater than 21 times were excluded as female kmers. Remaining kmers were then queried against the remaining date palm males and females as follows: greater than or equal to three in Zaghloul and Hayani Males were kept while less than or equal to nine or eight for Zaghloul and Hayani Females, respectively were discarded. As previously, the kmer count cutoffs were chosen to maximize the chance of obtaining kmers unique to males as demonstrated by their presence at half the coverage of the main kmer peak (hemizygous Y chromosome) while avoiding lower frequency kmers that are due to sequencing errors. This resulted in a set of kmers present in all males but absent in all females studied here.

The files of male-specific kmers for both the date palm and the phoenix species were queried against both alleles of the 10X genomics phased male assembly of the Deglet Noor male using BOWTIE v0.12.8 (Langmead et al., 2009). The numbers of male-specific kmers matching both alleles for each contig were documented. To identify contigs that are part of the Y chromosome we searched for contigs with at least 2.5X difference in numbers of male-specific kmers matching either assembled allele of the same contig. Further cutoffs were used as described below. To ensure a fair analysis of kmer density, we excluded the length of ambiguous and gap bases (N’s) from the total for all contigs. For previously sequenced BAC contigs we also excluded repeat (masked) bases from length. This was important as the BACs were sequenced in their entirety with Pacific Bioscience sequencing technology and so contained significant stretches of resolved repeats that the short-read 10X Genomics contigs would not resolve. To identify levels of divergence within the sex-determination region that could indicate recombination strata, we sorted contigs by the density of male-specific kmers and plotted the ratio between the average of the leading five and trailing five contigs male-specific kmer density. Peaks in this plot revealed significant changes in the density of male-specific kmers.

Putative syntenic regions were detected by BLASTX comparisons with the oil palm annotated reference proteomic sequences (GCF_000442705.1), using BLAST 2.9.0+. Only the first hit/best hit in oil palm was considered when assuming a conserved order of transcripts between both species. Collinearity between contigs and oil palm was confirmed by BLASTN comparisons with the recently released oil palm assembly (BioProject PRJNA636092).

## Results

By collecting kmers present in all three males and absent in all three females of the date palm cultivars used in this study we expected to identify Y chromosome specific polymorphisms that would allow the separation of X and Y linked contigs in a phased genome assembly. The 10X Genomics phased assembly of Deglet Noor male contained a total of 558 Mb in 51,834 contigs with an N50 length of 267 kb.

**FIGURE 3:**
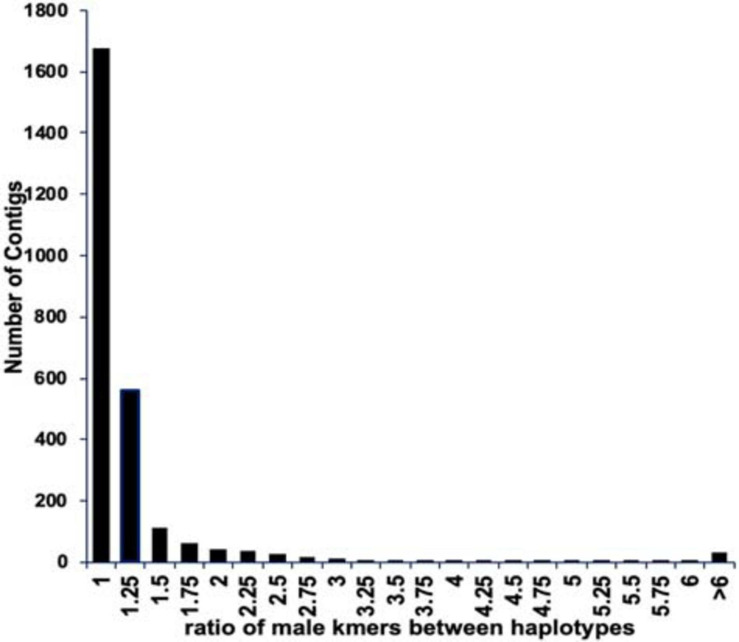
The ratio of male-specific kmers between both haplotypes of the phased Deglet Noor male assembly. Most contigs did not show a difference in the numbers of male-specific kmers matching each contig suggesting a lack of phasing separation or that the kmers are not truly Y chromosome specific. Contigs with greater than 2.5X difference in numbers of male-specific kmers between the two phases were considered for future analysis.

We found 6,778 contigs that contained matches to the presumed male-specific kmers, however in most cases there was no difference between the numbers of kmers matching the two haplotypes of a contig (Figure 3). To identify those contigs with well phased alleles separating the X and Y chromosome, we selected only those contigs with a difference between the two phased haplotypes of at least 2.5X resulting in 125 contigs spanning approximately 45 Mb (Supplementary Information). Furthermore, we selected those contigs with a density of at least one male-specific kmer per 2 kb (excluding ambiguous N bases) and at least 30 male-specific kmers in the scaffold. This resulted in a total of 60 scaffolds spanning 11.7 Mb (10.6 Mb excluding gaps and ambiguous bases) (Supplementary Table 1). For further analysis we included the previously assembled BAC contigs we identified in the genus-wide male-specific region (Torres et al., 2018). That increased the total to 66 scaffolds spanning a total of 13.6 Mb.

**FIGURE 4:**
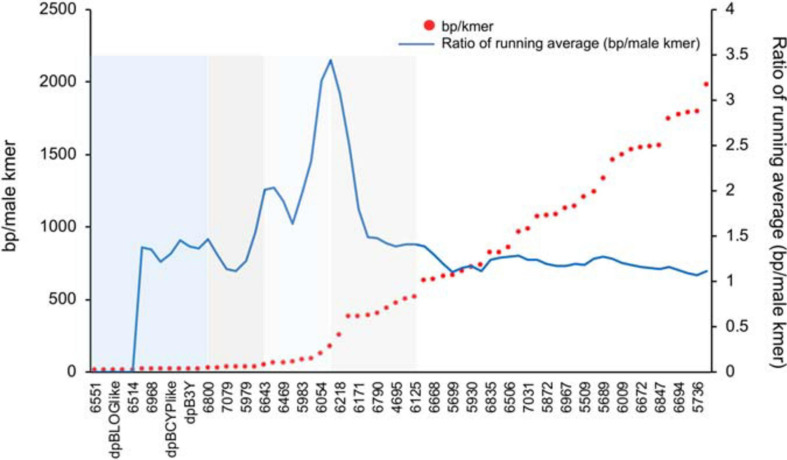
A plot of male-specific kmer density in contigs with significant differences between each assembled haplotype for three date palm male/female combinations. The density on the haplotype with the most kmers is plotted as base pairs of sequence per male-specific kmer. To identify regions of significant changes in the density, the running average of the leading five to trailing five contigs male-specific kmer density is plotted.

When the selected contigs were sorted by male-specific kmer density there was a generally smooth decrease in density except in between some contigs where a stepped pattern (Figure 4) was observed with an overall increase in distance between male-specific kmers (decrease in male-specific kmer density). To better locate the dividing point between contigs involved in these stepped shifts of density we plotted the ratio of the average density of the leading five vs. trailing five contigs (Figure 4). Peaks on this plot identified multiple shifts where the trailing five contigs had higher average male-specific kmer densities vs. the leading five contigs. These regions may suggest a possible event such as an inversion or translocation that resulted in a shared density of male-specific kmers among the contigs of that region. Based on the observed shifts in male kmer density, we grouped the 66 scaffolds into five groups or kmer density blocks (KDBs) (Supplementary Table 1).

**FIGURE 5:**
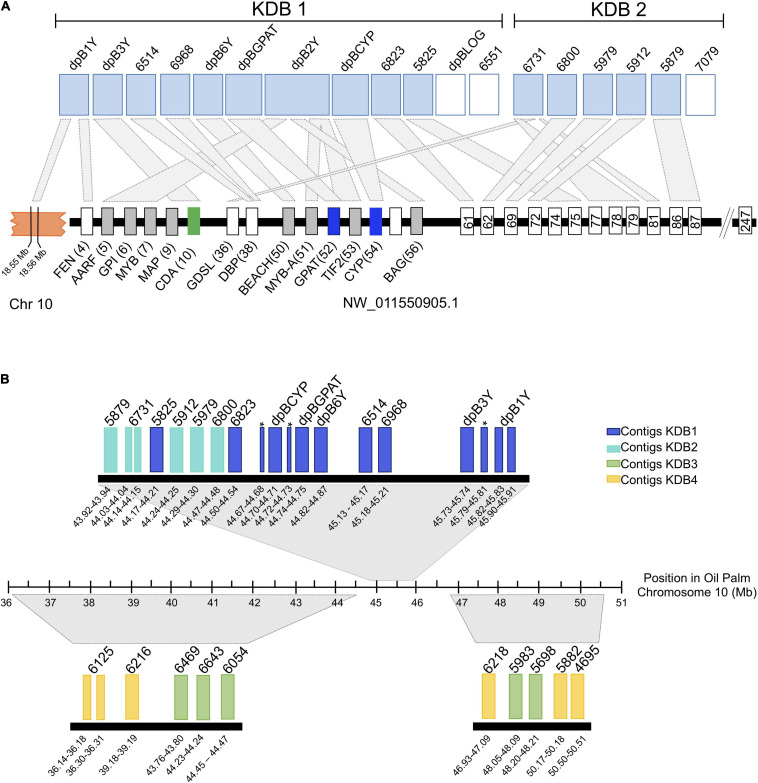
Alignment of the date palm male-rich kmer contigs to the current oil palm reference genome from kmer density blocks 1 and 2 **(A)** and all with significant alignment against the new oil palm assembly **(B)**. For panel **(A)** Rectangles at the top represent date palm contigs with high similarity (light blue) or no similarity with any oil palm transcript (white). Black rectangles represent contigs with similarity to other oil palm chromosomes The smaller rectangles at the bottom represent oil palm genes and the numbers indicate their predicted position within the scaffold. Homology between date palm contigs (top) and oil palm (bottom) is represented by light-gray rectangles. The orange block represents chromosome 10 and the numbers indicate approximate locations of the transcripts with homology to date palm contigs. For panel **(B)** the rectangles represent date palm contigs with high similarity with oil palm chromosome 10 (GeneBank accession GK000085.1). Large regions of homology between date palm contigs and oil palm are represented by gray trapezoids. The numbers below each contig indicate their approximate location in chromosome 10. The dark blue rectangles represent contigs within kmer density block 1 (KDB1). Lighter blue, green and yellow rectangles represent contigs within KDB2, KDB3, and KDB4, respectively. Asterisks appear next to dpB2Y alignments that split across the region. KDB, Kmer Density Block. FEN: LOC105059737; AARF: LOC105059738; GPI: LOC105059739; MYB: LOC105059740; MAP: LOC105059742; CDA: LOC105059743; GDLS: LOC105059768; DBP: LOC105059770; BEACH: LOC105059782; MYB-A: LOC105059783; GPAT: LOC105059961; TIF2: LOC105059784; CYP: LOC105059962; BAG: LOC105059785. Gene 61: LOC105059787; Gene 62: LOC105059788; Gene 63: LOC105059789; Gene 69: LOC105059792; Gene 74:LOC105059800; Gene 75: LOC105059801; Gene 77: LOC105059803; Gene 78: LOC105059804; Gene 79: LOC105059805; Gene 81: LOC105059809; Gene 86: LOC105059813; Gene 87: LOC105059814; Gene 92: LOC105059820.

Ordering of the KDBs was conducted solely on shifts in male-specific kmer density so next we looked to what synteny might reveal of the KDB structures. Syntenic analysis with the closely related monoecious oil palm indicated that contigs in the highest density KDB (KDB1) are associated with same locus that we previously determined contains the male-specific genes, an unplaced scaffold (NW_011550905.1) that spans 6.3 Mb and is predicted to contain 247 genes (Figure 5A). KDB 1 is composed by 12 contigs, spanning 2.08 Mb. Three of these contigs contain CYP703, GPAT3, and LOG, the genes that are completely absent in all female Phoenix species. Two other contigs, dpB3Y and dpB2Y, were also previously identified as important in the initial deletions and inversion events associated with recombination suppression between X and Y chromosomes in Phoenix (Torres et al., 2018). From the remaining seven contigs, six showed high similarity to different transcripts in the same locus, upstream and downstream of the inversion/deletion area, with one of them (dpB1Y) also showing similarity to a gene located in chromosome 10 in oil palm (Figure 5A).

KDB 2 is composed by six contigs spanning 406 kb, with five of them showing similarity to ten transcripts in the oil palm unplaced scaffold (Figure 5A). Using a recently released version of the oil palm genome that places the NW_011550921.1 on chr10 (Ong et al., 2020) we were able to compare our results in more detail. The date palm KDBs showed similarity to the new chr10 between Mb 36 and 51. KDB 3 (Figure 5B) is composed by seven contigs spanning 319 Kb, with five contigs showing collinearity with chromosome 10. Our analysis also indicated that one contig from KDB 1, one from KDB 2 and two from KDB 3 did not show similarities to any oil palm transcript.

KDB 4 spans 10 contigs, covering a region of 3.1 Mb (Figure 5B). Four contigs in this newer group are highly syntenic with chromosome 10 in oil palm, while the remaining six contigs showed similarities to transcripts located in chromosomes 2, 3, 13, and the unplaced scaffold NW_011550921.1 (Supplementary Table 1). KDB 5 contains 31 contigs and spans 7.6 Mb, approximately half of the Y-specific region identified in this study. Synteny of this large region with oil palm was harder to establish, but many contigs showed similarities to contiguous genes in chromosomes 3 and 10 and less frequently to chromosome 2 (data not shown).

## Discussion

We recently sequenced and examined the genomes of male and female individuals representing all 14 species of the genus Phoenix, and identified 1,653 male-specific kmers present in 13 males and absent in all 14 female species. This analysis allowed the identification of four genes, CYP703, GPAT3, LOG, absent from all Phoenix females, and cytidine deaminase, which remains in both X and Y-linked regions (Figure 1). Because three of these genes were also mapped to a single locus in the closely related monoecious oil palm, it is likely this area represents the initial steps that gave rise to dioecy in the genus while subsequent alterations around it established the formation of X and Y chromosome structures in date palm.

We analyzed three date palm male/female combinations from the North African subpopulation of date palm. By focusing on North African cultivars we hoped to better identify evolutionary changes specific to the Y chromosome at the most resolved level possible being the date palm subpopulation level. Indeed, previous work has shown possible structural differences between Y chromosomes of the two date palm subpopulations that could hide evolutionary strata were the analysis combined (Cherif et al., 2013).

A Y chromosome with consistently increasing male specific kmers would be expected under a model of continuous Y chromosome degradation through decreased recombination. However, if there have been events of large-scale recombination suppression during the Y chromosome evolution, these will show as blocks of changes in male-specific kmer densities. We chose not to use a sliding window approach on larger scaffolds but rather consider each scaffolds density under the assumption that large kmer density shifts would be difficult to detect on a fine scale. While these shifts could be a result of significant gaps in the assembled Y chromosome resulting in an apparent jump in male-specific kmers rather than the expected smooth increase, they offer regions to focus analysis on for possible chromosomal rearrangements during the evolution of the date palm Y chromosome.

While we observed multiple steps in the density of male-specific kmers across the selected scaffolds, the largest jump appears in the vicinity of scaffolds 6054, 6216, and 6218 (Figure 4). The trailing five scaffolds of 6216 contain an average of 85 bp between male-specific kmers while the leading five scaffolds contain and average of approximately 260 bp between kmers. This more than threefold drop in density could indicate a large-scale structural change and warrants further investigation. Use of the newly released oil palm chr10 (Ong et al., 2020) confirms the general structure of the date palm sex-linked contig placement with a central core of high male-specific SNP density slowly dissipating away from this region. With this more detailed understanding of the date palm Y chromosome we hope to guide further studies of the major structural changes that have occurred during sex-chromosome development in date palm.

## Data Availability Statement

The datasets analyzed for this study can be found in NCBI SRA trace archive using accessions: SRR6439416, SRR6439410, SRR6716026, SRR6716027, SRR6716028, and SRR6716029. The 10X Genomics phased contigs are included in the Supplementary Information File.

## Author Contributions

MT conducted evolutionary analysis of date palm sequences and helped write the manuscript. YM directed and analyzed 10X phased and Illumina sequencing of the genomes. SY analyzed genomic data. KS analyzed the data and wrote the manuscript. JM envisioned the project, analyzed data, and wrote the manuscript. All authors contributed to the article and approved the submitted version.

## Conflict of Interest

The authors declare that the research was conducted in the absence of any commercial or financial relationships that could be construed as a potential conflict of interest.

## Supplementary Material

Supplementary Table 1 An excel file containing information about contigs that passed all filters in this study including which Kmer Density Block they are assigned to.

Supplementary Information A text file of FASTA sequence entries for all phased scaffolds analyzed here. A contig contains an “Xchr” or “Ychr” in the title to denote which allele it is predicted to be from.
